# Effect of Diarrheal Illness During Pregnancy on Adverse Birth Outcomes in Nepal

**DOI:** 10.1093/ofid/ofz011

**Published:** 2019-01-14

**Authors:** Kira L Newman, Kathryn Gustafson, Janet A Englund, Amalia Magaret, Subarna Khatry, Steven C LeClerq, James M Tielsch, Joanne Katz, Helen Y Chu

**Affiliations:** 1 Department of Medicine, University of Washington, Seattle; 2 Center for Clinical and Translational Research, Seattle Children’s Research Institute, Seattle, Washington; 3 Nepal Neonatal Intervention Project-Sarlahi, Nepal; 4 Department of Global Health, George Washington University Milken Institute School of Public Health, Washington, District of Columbia; 5 Department of International Health, Johns Hopkins University Bloomberg School of Public Health, Baltimore, Maryland

**Keywords:** birth outcomes, diarrhea, Nepal, pregnancy, small for gestational age

## Abstract

**Background:**

Adverse birth outcomes, including low birthweight, small for gestational age (SGA), and preterm birth, contribute to 60%–80% of infant mortality worldwide. Little published data exist on the association between diarrhea during pregnancy and adverse birth outcomes.

**Methods:**

Data were used from 2 community-based, prospective randomized trials of maternal influenza immunization during pregnancy conducted in rural Nepal from 2011 to 2014. Diarrheal illnesses were identified through longitudinal household-based weekly symptom surveillance. Diarrhea episodes were defined as at least 3 watery bowel movements per day for 1 or more days with 7 diarrhea-free days between episodes. The Poisson and log-binomial regression were performed to evaluate baseline characteristics and association between diarrhea during pregnancy and adverse birth outcomes.

**Results:**

A total of 527 of 3693 women in the study (14.3%) experienced diarrhea during pregnancy. Women with diarrhea had a median of 1 episode of diarrhea (interquartile range [IQR], 1–2 episodes) and 2 cumulative days of diarrhea (IQR, 1–3 days). Of women with diarrhea, 85 (16.1%) sought medical care. In crude and adjusted analyses, women with diarrhea during pregnancy were more likely to have SGA infants (42.6% vs 36.8%; adjusted risk ratio = 1.20; 95% confidence interval, 1.06–1.36; *P* = .005). Birthweight and preterm birth incidence did not substantially differ between women with diarrhea during pregnancy and those without.

**Conclusions:**

Diarrheal illness during pregnancy was associated with a higher risk of SGA infants in this rural South Asian population. Interventions to reduce the burden of diarrheal illness during pregnancy may have an impact on SGA births in resource-limited settings.

Adverse birth outcomes, including low birthweight (LBW), small for gestational age (SGA), and preterm birth, contribute to 60%–80% of infant mortality worldwide [1, 2]. The impact of diarrheal diseases on children and infants is well established. Globally, diarrheal illness is responsible for 6% of overall adult mortality [3] and is a major cause of morbidity. Studies of diarrhea during pregnancy (1) have examined only women who were hospitalized for diarrheal illness or (2) have focused on specific etiologies such as listeria or typhoid, which are known to cause severe disease in pregnant women [4–9]. Community-based studies that have included women who were not hospitalized have generally examined infant neurocognitive outcomes and growth [4, 10, 11].

Data from hospitalized women with diarrhea indicate that multiple different pathogen types can cause severe adverse birth outcomes, including premature rupture of membranes, miscarriage, and neonatal infection [5–7, 12]. However, these studies fail to capture the denominator of women who experience acute diarrheal illness but do not seek care or are less critically ill. In the absence of recent data regarding the incidence of diarrhea during pregnancy, it is difficult even to estimate the potential magnitude of any adverse birth outcome that diarrhea might cause. This is particularly important in low-income countries where adverse birth outcomes have significant implications for neonatal morbidity and mortality and where the incidence of diarrhea is high [13, 14]. The objective of this study was to assess the incidence of diarrhea during pregnancy and evaluate the association between diarrhea during pregnancy and adverse birth outcomes in rural southern Nepal.

## METHODS

We used data from 2 community-based, prospective randomized trials of maternal influenza immunization of 3693 pregnant women and their infants conducted in rural Nepal from 2011 to 2014. Verbal informed consent was obtained from women at enrollment in surveillance and again at time of immunization. Detailed methods and results of these clinical trials have been published elsewhere [15]. In brief, pregnant women who were 15–40 years old and 5–34 weeks’ gestation were enrolled and had data collected from the time of enrollment through 6 months after birth. Sociodemographic characteristics of the women and their households were collected, and symptom data were collected weekly through home visits by trained field staff who asked about daily symptoms and signs over each of the past 7 days. These included watery stools, nausea, vomiting, abdominal pain, fever, and myalgia. Pregnancy-related morbidity as well as serial weight and blood pressure measurements were assessed through monthly home visits. A home visit after birth was conducted to collect birthweight and other data about birth outcomes.

For this secondary analysis, diarrheal illness episodes were defined as at least 3 watery stools per day for 1 or more days [16] with 7 diarrhea-free days between episodes. Maternal smoking status was based on whether cigarettes or bidi (hand-rolled cigarettes) were smoked within 30 days of enrollment. Ethnic group was dichotomized as Pahadi (traditionally from the hills of Nepal) or Madheshi (traditionally from the plains). Household density was defined as the number of persons per room, excluding storerooms and kitchens. Care seeking was defined as having seen a local physician, health worker, or traditional healer (dhami-jhankri) for symptoms. Maternal body mass index (BMI) was ascertained during early pregnancy at the time of enrollment. Symptom data were missing completely for 2 study subjects. Of 590 867 days of follow up, diarrhea symptoms were missing or invalid for 113 340 days (19.2%). The median frequency of missing symptom data was 14.6%, and 228 women (6.2%) were missing over 50% of symptom data. Days with missing data were treated as days without diarrhea.

The primary birth outcomes assessed were birthweight and gestational age at birth. Low birthweight was defined as <2500 grams. Small for gestational age was defined using INTERGROWTH-21 standards [17]. Preterm birth was defined as birth before 37 weeks completed gestation. For bivariate analysis of the association between baseline variables and incidence of diarrhea, Poisson regression was used with an offset for follow-up time. Adjusted associations between diarrhea during pregnancy and the binary outcomes of LBW, SGA, and preterm birth were assessed using log-binomial regression models. Adjusted models included presence of children under 5 years of age in the household, latrine type, running water in the household, household electricity, maternal smoking, Brahmin status, and ethnicity. For the adjusted model of preterm birth, follow-up time was included as a covariate and an interaction term with diarrhea to account for time at risk to experience an episode of diarrhea. The preterm birth model did not converge when all the variables were included, so the final adjusted model was limited to diarrhea, follow-up time, Brahmin status, presence of children under 5 in the household, and household running water.

All analyses were performed using R version 3.5.0 (R Foundation for Statistical Computing) in RStudio Version 1.1.453 (RStudio, Inc.). The Johns Hopkins University Bloomberg School of Public Health, Cincinnati Children’s Hospital, the Institute of Medicine at Tribhuvan University, and the Nepal Health Research Council institutional review boards approved this study and the randomized controlled trials. The primary trial was registered with ClinicalTrials.gov (Trial no. NCT01034254).

## RESULTS

### Incidence and Risk Factors for Diarrhea

The trials enrolled 3693 eligible women between April 2011 and September 2013, and weekly household surveillance visits continued until the end of May 2014. Median follow-up time was 175 days (interquartile range [IQR], 133–206) among women with diarrhea and 161 days (IQR, 120–199) among women without diarrhea.

**Figure 1.**
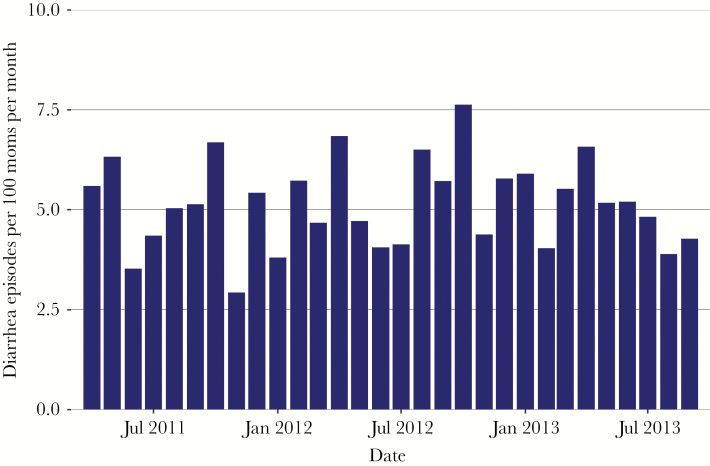
Histogram of diarrhea episodes per 100 pregnant women per month during study period. Final 3 months of surveillance not shown because fewer than 20 mothers were still being followed.

**Figure 2.**
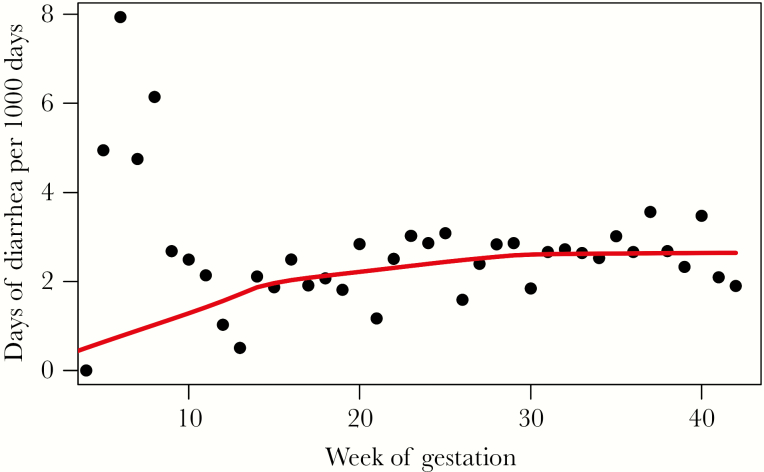
Frequency of diarrhea during pregnancy by gestational age. Black dots indicate days of diarrhea for women at each week of gestation weighted by the number of women enrolled in the study at each time point. Red line indicates trend.

**Figure 3.**
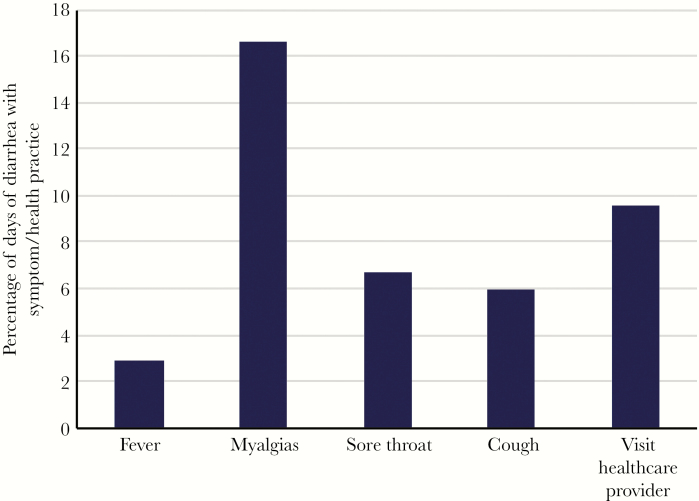
Bar chart of concurrent symptoms and health practices of pregnant Nepali women with diarrhea.

Overall, 527 (14.3%) of women had 1 or more episodes of diarrhea during pregnancy (Table 1). The incidence of diarrhea was 848.7 cases/1000 person-years among pregnant women (95% confidence interval [CI], 803.8–893.6). Incidence was relatively constant throughout the study period and did not vary by season (Figure 1). The median number of diarrhea episodes during pregnancy was 1 (IQR, 1–2), with a median total number of days of diarrhea during pregnancy of 2 (IQR, 1–3). When adjusted for the number of women enrolled in the trial, the incidence of diarrhea was relatively stable during the second and third trimesters (Figure 2). Of women who had diarrhea during pregnancy, 28 (5.3%) had 1 or more episodes of diarrhea with fever, and 85 (16.1%) sought medical care for at least 1 episode of diarrhea (Figure 3). Influenza vaccine receipt was not associated with the incidence of diarrhea.

**Table 1.** Demographic and Household Characteristics of Rural Nepali Women With and Without Diarrhea During Pregnancy

Variable	Whole Cohort	No Diarrhea	Diarrhea	*P* Value
	n = 3693	n = 3164	n = 527	
Maternal age at delivery (mean, SD)	23.00 (4.73)	22.96 (4.73)	23.23 (4.72)	.306
Influenza vaccine recipient (n, %)	1847 (50.0)	1598 (50.5)	247 (46.9)	.157
Maternal BMI (mean, SD)	21.01 (2.86)	21.06 (2.86)	20.72 (2.82)	.844
<16	45 (1.2)	37 (1.2)	8 (1.5)	
16–18.5	603 (16.3)	497 (15.7)	106 (20.1)	
18.5–25	2724 (73.8)	2347 (74.2)	375 (71.2)	
>25	310 (8.4)	275 (8.7)	35 (6.6)	
Nulliparous (n, %)	1548 (42.0)	1322 (41.8)	225 (42.9)	.546
Mother smokes (n, %)	114 (3.3)	95 (3.2)	18 (3.6)	.401
Mother literate (n, %)	2095 (60.3)	1793 (60.3)	301 (60.4)	.733
Brahmin (n, %)	382 (10.6)	315 (10.2)	67 (13.0)	.060
Madeshi (n, %)	1555 (43.3)	1350 (43.9)	204 (39.6)	.185
Household Characteristics				
Latrine (n, %)	1759 (49.0)	1516 (49.3)	242 (47.1)	.227
Electricity (n, %)	3230 (89.9)	2761 (89.8)	468 (91.1)	.648
Running water (n, %)	3037 (84.6)	2613 (85.0)	422 (82.1)	.101
Indoor cookstove (n, %)	2937 (81.7)	2508 (81.4)	427 (82.9)	.147
Any smokers in household (n, %)	1677 (46.9)	1453 (47.5)	222 (43.4)	.101
Persons per room (mean, SD)	4.10 (3.10)	4.10 (3.16)	4.04 (2.68)	.770
Child under 5 years old (n, %)	2309 (62.5)	1959 (61.9)	348 (66.0)	.030

Abbreviations: BMI, body mass index; SD, standard deviation.

NOTE: *P* values determined using bivariate Poisson regression with follow-up time as offset. Columns do not sum to total cohort n because 2 women from original cohort had no diarrhea symptom data available and were excluded from further analyses.

Women who experienced diarrhea during pregnancy were more likely to have other children under 5 years old living in their household than women who did not experience diarrhea (66.0% vs 61.9%, *P* = .030). There were no differences in maternal BMI, ethnicity, class, literacy, or smoking status by whether women had diarrhea in pregnancy. There were also no household differences other than the presence of children under 5 years old.

### Association With Birth Outcomes

All but 6 women with diarrhea during pregnancy delivered live infants (521, 98.9%), which was a similar proportion to women without diarrhea, among whom 97.9% had live births (n = 3097). The median gestational age of infants in both groups was 39.7 weeks (IQR, 38.3–40.9 weeks), and the median birthweight in both groups was 2790 grams (IQR, 2500–3078 grams). Overall, the prevalence of LBW was 24.8% (n = 679, 24.8% of births among women without diarrhea vs 25.0% among women with diarrhea), the prevalence of preterm birth was 13.4% (n = 492, 13.5% of births among women without diarrhea vs 12.3% among women with diarrhea), and the prevalence of SGA was 37.7% (n = 1038, 36.8% of births among women without diarrhea vs 42.6% among women with diarrhea).

**Figure 4.**
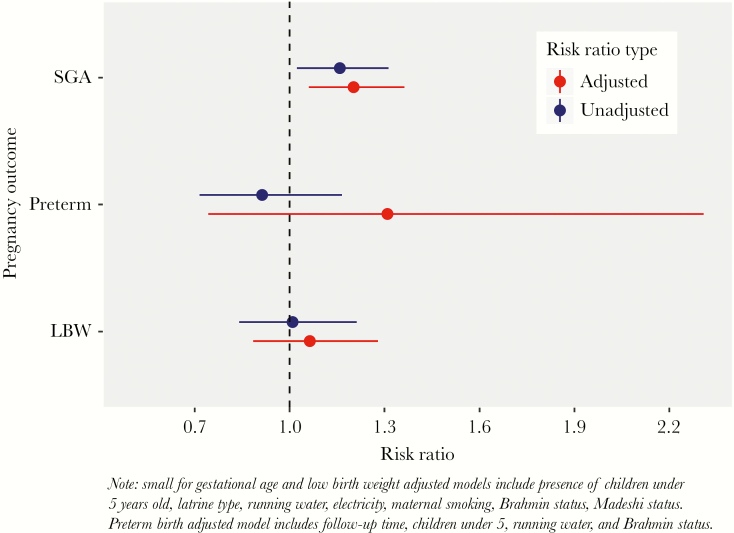
Unadjusted and adjusted risk ratios for the association between diarrhea during pregnancy and adverse birth outcomes among women in Nepal. LBW, low birthweight; SGA, small for gestational age.

In unadjusted analyses, women with diarrhea during pregnancy were 1.16 times more likely to deliver a SGA infant compared with women without diarrhea during pregnancy (95% CI, 1.02–1.31) (Figure 4). No association was found between diarrhea during pregnancy and LBW (risk ratio [RR] = 1.01; 95% CI, 0.84–1.21) or preterm birth (RR = 0.92; 95% CI, 0.72–1.17).

In the fully adjusted model, women with diarrhea during pregnancy were 1.20 times more likely to have a SGA infant compared with women without diarrhea during pregnancy (95% CI, 1.06–1.36) (Figure 4). No association was found between diarrhea during pregnancy and LBW (RR = 1.06; 95% CI, 0.88–1.28) or preterm birth (RR = 1.31; 95% CI, 0.74–2.31) in the adjusted models, although there was a trend towards greater risk of both negative birth outcomes in the adjusted models compared with the unadjusted models.

## DISCUSSION

In this study, we performed intensive weekly community-based prospective surveillance for diarrheal illness during pregnancy in a large cohort of women in Nepal, a low-income country setting with high rates of adverse birth outcomes. Across 3 years, we found that diarrhea incidence was relatively high during pregnancy compared with contemporaneous data from Nepal’s general population, it was associated with having children under age 5 years in the household, and it increased the risk of having a SGA infant.

In 2012 in Nepal, the estimated annual prevalence of diarrheal disease among women aged 15–49 was 12.3%, and diarrhea was responsible for 3.7% of deaths in women of this age group [18]. Among the women in our study, the prevalence of diarrhea was higher (14.3% during the average of 6 months for which they were followed). We do not have data regarding the baseline prevalence of diarrhea during the study period for this region of Nepal where the study was conducted. However, this study suggests that pregnant women may have diarrhea at or exceeding the national rate for their age group.

Diarrhea during pregnancy increased the risk of having a SGA infant by approximately 20%. Compared with AGA infants, SGA infants are at increased risk for bronchopulmonary dysplasia [19], necrotizing enterocolitis [20], and mortality [13, 21]. Studies in Nepal have quantified the risk of mortality for SGA infants, finding that SGA infants had between a 40% and 200% higher risk of neonatal death than AGA infants [13, 21]. Given the increased risks that these infants face and the high prevalence of SGA births in our cohort and in other low- and middle-income countries [22], interventions that reduce the incidence of diarrhea during pregnancy may reduce the burden of adverse pregnancy outcomes. This finding may also be generalizable to other low-income countries with high rates of diarrheal disease and SGA birth. Further research should work on implementing water, sanitation, and hygiene interventions among pregnant women and tracking the impact on birth outcomes.

One of the important predictors of diarrhea was presence of a child under 5 years old in the household. In Nepal, children under 5 years old have a high prevalence of diarrhea [23], and it is a leading cause of childhood morbidity and mortality [24]. Children under 5 are at greater risk of infection with gastrointestinal pathogens than adults [25], and they may represent a source for infections among household members. Rotavirus is a common cause of severe diarrhea among children in Nepal [26], and the government of Nepal has recently begun to roll out rotavirus vaccine across the country [27]. Programs such as this, which prevent childhood diarrhea, may have additional benefits for other household members, including pregnant women.

A possible mechanism by which diarrhea during pregnancy may cause SGA infants may be through impaired nutrition. Undernutrition before and during pregnancy and poor dietary quality are associated with SGA birth [28–30]. In the low-income setting of rural Nepal, the effect of decreased nutrition even from a transient episode of diarrhea may be more pronounced given high baseline levels of acute and chronic malnutrition [13], and therefore it may increase the risk of SGA birth. Another possible mechanism is through infectious or inflammatory pathways that lead to impaired intrauterine growth. This has been demonstrated for malaria [31, 32], but no such studies exist for common diarrheal pathogens. Inflammation resulting from infection may also play a role through impaired growth pathways and reduced nutritional transfer from mother to fetus [11, 33, 34].

Strengths of our study include its size, design, and duration of follow up. Our study used data from a large cohort of women who were followed prospectively with weekly symptom reporting. Women were followed for a median of approximately 6 months during their pregnancies. Limitations of our study include the following: the lack of stool collection and therefore the absence of pathogen-specific diagnosis; the limitation to a single rural district, although its population was broadly representative in density and birth rate; the presence of missing data for some participants; and the use of weekly recall for symptom data collection. Missing data was treated as days without diarrhea, and this may have biased our estimates of the association between diarrhea and adverse pregnancy outcomes towards the null.

## CONCLUSIONS

Diarrhea is relatively common during pregnancy among women in rural Nepal. It is associated with SGA infants but not with preterm birth or LBW. Interventions to reduce the burden of diarrheal illness during pregnancy may have an impact on SGA births in resource-limited settings. Future work should expand to other populations to develop more comprehensive global data regarding the incidence and consequences of diarrhea among pregnant women from a range of geographic and socioeconomic backgrounds and to test targeted interventions to prevent diarrheal illness in this population.
